# Trends in Use of Referral Hospital Services for Care of Sick Newborns in a Community-based Intervention in Tangail District, Bangladesh

**Published:** 2006-12

**Authors:** Sanwarul Bari, Ishtiaq Mannan, Mohammed Anisur Rahman, Gary L. Darmstadt, M. Habibur R. Seraji, Abdullah H. Baqui, Shams El Arifeen, Syed Moshfiqur Rahman, Samir K. Saha, A.S.M. Nawshad Uddin Ahmed, Saifuddin Ahmed, Mathuram Santosham, Robert E. Black, Peter J. Winch, Bangladesh Projahnmo-II Study Group

**Affiliations:** ^1^ ICDDR,B, GPO Box 128, Dhaka 1000, Bangladesh; ^2^ Department of International Health, Bloomberg School of Public Health, Johns Hopkins University, Baltimore, MD, USA; ^3^ Department of Microbiology, Dhaka Shishu (Children's) Hospital, Dhaka, Bangladesh; * Projahnmo-II Study Group (in alphabetical order): Saifuddin Ahmed, A.S.M. Nawshad Uddin Ahmed, Nabeel Ashraf Ali, Tariq Anwar, Shams El Arifeen, Abdullah H. Baqui, Sanwarul Bari, Nazma Begum, Robert E. Black, Atique Iqbal Chowdhury, Sameena Chowdhury, Gary L. Darmstadt, A.K.M. Fazlul Haque, Quamrul Hasan, Ishtiaq Mannan, Dulal Poddar, Mohammed Anisur Rahman, Qazi Sadequr Rahman, Syed Moshfiqur Rahman, Samir K. Saha, Mathuram Santosham, M. Habibur R. Seraji, Ashrafuddin Siddik, and Peter J. Winch

**Keywords:** Delivery of healthcare, Health services, Care-seeking, Referral and consultation, Community health workers, Neonatal health, Maternal health, Bangladesh

## Abstract

The Projahnmo-II Project in Mirzapur upazila (sub-district), Tangail district, Bangladesh, is promoting care-seeking for sick newborns through health education of families, identification and referral of sick newborns in the community by community health workers (CHWs), and strengthening of neonatal care in Kumudini Hospital, Mirzapur. Data were drawn from records maintained by the CHWs, referral hospital registers, a baseline household survey of recently-delivered women conducted from March to June 2003, and two interim household surveys in January and September 2005. Increases were observed in self-referral of sick newborns for care, compliance after referral by the CHWs, and care-seeking from qualified providers and from the Kumudini Hospital, and decreases were observed in care-seeking from unqualified providers in the intervention arm. An active surveillance for illness by the CHWs in the home, education of families by them on recognition of danger signs and counselling to seek immediate care for serious illness, and improved linkages between the community and the hospital can produce substantial increases in care-seeking for sick newborns.

## INTRODUCTION

The timely and appropriate use of health services is crucial to reduce maternal and child mortality (1–4). Many lifesaving interventions, such as caesarean section, blood transfusion, oxygen, and intravenous antibiotics, can only be made available through health facilities. Evidence suggests that bringing women and children to facilities to receive these services is associated with reductions in mortality (1, 4). Countries that have achieved high rates of deliveries in facilities for basic or comprehensive essential obstetric care have witnessed significant and sustained decreases in maternal mortality (4), while countries, such as Sri Lanka, with elevated rates of care-seeking for sick children, have much lower rates of mortality among children aged less than five years (under-five mortality) than would be predicted from their per-capita income (1).

Numerous studies have examined community-based interventions to improve care-seeking and referral for maternal emergencies from the community to first- and second-level facilities (3–8). Murray and Pearson have recently published a systematic review and proposed a research agendum to identify how programmes can better promote timely and appropriate maternal referral (9). Sibley has reviewed the effectiveness of traditional birth attendants (TBAs) in promoting referral from the community to facilities offering emergency obstetric care (10). Much less is known about referral of sick newborns from the community to health facilities. While several studies have examined referral of children, aged less than five-years, from first-level to second-level facilities (11–13), few studies have specifically examined referral from the community to facilities (14), and even less is known about referral of newborns from the community to facilities.

Many factors affecting referral and care-seeking for maternal conditions from the community to health facilities could be applicable to promoting care for sick newborns. First, there is a need to act rapidly for certain maternal and newborn conditions, such as severe postpartum haemorrhage and birth asphyxia. So, research needs to examine not only recognition of danger signs, but also how long it takes for recognition of the problem to occur. The constraints on rapid action by families, such as distance, poor conditions of road, lack of transport, and lack of money, are common to both. Macintyre and Hotchkiss have developed a conceptual framework of factors at the individual, household, and community levels affecting referral in Africa which is equally applicable to other regions of the world (15). Second, in both mothers and newborns, many important causes of morbidity and mortality have signs and symptoms that “lie on a continuum, from normal to abnormal” (16). For such signs and symptoms, there can be both over-reporting of non-serious conditions, such as transient tachypnoea of the newborn or upper respiratory viral infection, or under-reporting of truly serious conditions, as happens when families fail to recognize excessive loss of maternal blood after delivery (16). Finally, patterns of decision-making within the household may lead to a significant delay, especially when permission of the husband is needed before seeking care but he is away, or when it is not clear to the family where to seek care from among a range of formal and informal sector providers (17–19).

While there is much to learn from previous research on care-seeking and referral for older children, there are also obstacles to promoting early and appropriate care that are specific to newborns. In many cultures, families practise a period of postpartum confinement of both mother and newborn lasting from one to six week(s) or more (20). Additional efforts may be needed during this period to convince families to seek care outside the home. Sometimes, it is also difficult to detect danger signs in sick newborns, and families may not understand the significance of signs, such as hypothermia, feeding problems, or lethargy (21).

This paper describes the trends in compliance by families with referrals when sick newborns were identified by community health workers (CHWs) and were referred to the Kumudini Hospital for care. In this project, families are educated about danger signs, and the CHWs visit the home during the postpartum period and examine newborns for any signs indicating the need for urgent medical care. Actions to increase the rate of compliance with referrals and their effects are also described.

## MATERIALS AND METHODS

### Study site

The Projahnmo-II Project in Mirzapur upazila (sub-district) of Tangail district in central Bangladesh is implementing an intervention aimed at improving maternal and newborn-care practices and care-seeking for maternal and newborn conditions through: (a) behaviour change communication, (b) identification and referral of sick newborns in the community, and (c) strengthening of neonatal care in health facilities. The cluster-randomized trial has two arms: an intervention arm with CHWs delivering a package of maternal and newborn-care interventions in the home and a comparison arm. Mirzapur upazila has 13 unions, with a population of around 24,000 each; of these, six were randomly allocated to each study arm, excluding the one urban union. Given the need to have sick neonates assessed and treated in the hospital to identify aetiology of infections, a major focus of the problem is to identify barriers to care-seeking and design of strategies to address those barriers. The Kumudini Hospital, a 750-bed private hospital run by the Kumudini Welfare Trust, is situated at the centre of the project area. The hospital has a large paediatric unit (total 65 beds, including one separate neonatal ward) with two full-time consultants and 7–8 regular physicians.

### Description of the intervention model

In the Projahnmo-II project, 36 CHWs were recruited and provided one month of initial training to equip them to provide a package of maternal and newborn care. These CHWs had a minimum of 10th grade education and resided in the population they would serve. Each CHW was responsible for about 4,000 people. The CHWs carried out bi-monthly pregnancy surveillance and registration of married women of reproductive age (MWRA) and made home-visits in the third and the eighth month of pregnancy to counsel families on birth and neonatal care preparedness (BNCP). After delivery, the CHWs made home-visits to promote evidence-based domiciliary newborn care and to identify and refer sick newborns and mothers on day 0 (day of birth), 3, 6, and 9. Improvements were made in maternal and neonatal healthcare at the designated referral facility (Kumudini Hospital), and the CHWs facilitated referrals of sick newborns they identified during home-visits to this hospital. For families that refused referral and for babies who had at least one danger sign of very severe disease, or any two from possible very severe disease, the CHWs carried out home-treatment of cases with suspected serious infection with oral co-trimoxazole. The Field Supervisors supervised the daily activities of CHWs in the community and also reviewed the key performance and process indicators relating to coverage and quality every fortnight and provided feedback to the CHWs.

**Fig. 1.**
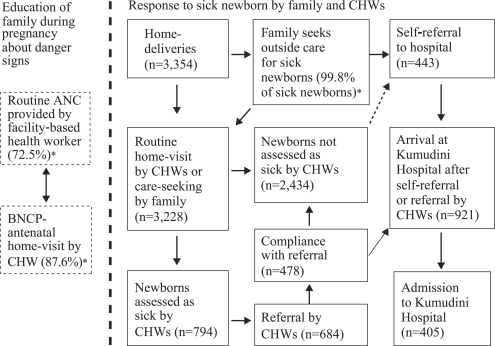
Intervention model: promotion by community health workers of care for sick newborns at Kumudini Hospital between April 2004 and September 2005 (Table 1)

Figure 1 is a model that shows how the intervention package helps promote hospital care for sick newborns. During the antenatal period, families were educated about signs indicating the need to seek care and sources of care by health workers during antenatal care visits at health facilities and by CHWs during antenatal home-visits (left side of Fig. 1). This should lead to an increased recognition by families of danger signs in newborns. This, in turn, should lead them either to seek care directly (self-referral) from the Kumudini Hospital and other appropriate sources of care, or notify the CHW to come to the house and assess the child. The CHW may also come in contact with a sick newborn during the course of her routine postnatal home-visits, and all findings are recorded in a Visit Record Form (VRF). The criteria for referral of sick newborns are: (a) in the case of birth asphyxia: if breathing difficulty continues 20 minutes after birth despite resuscitation efforts; (b) if there is one or more signs/symptoms of very severe disease, such as fever or lethargy; (c) jaundice—any where in the body within 24 hours of birth; (d) eyes discharging pus (possible gonococcal eye infection); and/or (e) diarrhoea with blood in stool and/or dehydration.

Dotted arrow indicates action not anticipated in the intervention model
*Coverage estimates from the second adequacy survey, September 2005
Sources of data: Project management information system (records maintained by CHWs and hospital registers) and household survey (second adequacy survey in September 2005)
ANC=Antenatal care;
BNCP=Birth and neonatal care preparedness;
CHWs=Community health workers

A standard algorithm adapted from integrated management of childhood illness (IMCI) for use in the community by the CHWs is followed to assess and classify sickness in newborns. If the newborn requires referral, the CHW facilitates referral to the Kumudini Hospital. The community-based system of facilitated referral was in place by 19 February 2004 and initially consisted of the following elements, in addition to home-visits by the CHWs: (a) referral slips for the CHWs to fill in when referring a sick newborn to the hospital; (b) Birth and Neonatal Care Preparedness (BNCP) Cards, with an identification number, are supplied to families during antenatal home-visits by the CHWs. Families can carry the BNCP Card with them to the Kumudini Hospital in the event that the CHW is not available to issue a referral slip to the family when the newborn falls ill; (c) a referral-tracking form; (d) free inpatient care at the Kumudini Hospital for newborns arriving with a referral slip; (e) a system of emergency transport; and (f) training of TBAs, so that they could also promote referral to hospital for newborns with danger signs.

If referral to the Kumudini Hospital fails, additional follow-up visits in the home are made to follow the condition of the child and treat presumptively with antibiotics, if appropriate. The CHW makes a final visit—called a graduation visit—as soon as the baby crosses 28 days of life. At that time, the completed VRF goes to the Mirzapur field office for review and is then sent to the main project office in Dhaka for data entry and further review.

### Modifications to the intervention model

The CHWs helped families to arrange transportation, convinced family members to have the newborn treated outside the home, and provided a transport allowance to the poorest families to reach the Kumudini Hospital. After three months of initial implementation of the programme, further changes were made to improve coverage with home-visits and compliance with referrals. A referral-tracking form was introduced in April 2004, and every two weeks, the number of newborns referred and the outcome of referral were reviewed in a meeting with the supervisors and CHWs. In early 2005, a decision was made to emphasize more the management and referral of birth asphyxia and low-birth-weight newborns. The CHWs started to use digital weighing machines in February 2005 to obtain the weight of the newborn at first contact. In April 2005, the CHWs received refresher training on how to counsel families during antenatal home-visits on low birth-weight and birth asphyxia.

### Monitoring of functioning of the referral system

Data are presented from the project management information system (MIS) for the April 2004–September 2005 period, which includes the records maintained by the CHWs and registers maintained in the referral hospital (Kumudini Hospital). In addition, in-depth interviews were undertaken at baseline and during implementation to investigate the factors affecting compliance. Results of the interviews with parents appearing at the Kumudini Hospital and from those who did not comply with referral advice were shared with the CHWs and Field Supervisors to identify ways to improve counselling on referral.

### Sampling and sample size

A baseline household survey among recently-delivered women was conducted in early 2003. Demographic and socioeconomic information of the listed households was collected, and information on birth-history and neonatal mortality was collected from all 14,526 women who had a pregnancy outcome in the 36 months preceding the survey. Information on knowledge, practices, and coverage of pregnancy, childbirth, and postpartum/postnatal care of mothers and newborns was collected from a randomly-selected sub-set of 4,611 recently-delivered women who had a pregnancy outcome in the last one year.

Two interim household adequacy surveys were conducted in January and September 2005 to measure the adequacy of project inputs and selected process indicators to assess progress in early changes in behavioural patterns, including care-seeking. Identified through a systematic random-sampling method, in total, 1,200 women who had a pregnancy outcome in the last 7–8 months in each of the intervention and comparison arms were interviewed in each of the interim household surveys.

Eighty-four randomly-selected parents arriving in the Outpatient Department of the Kumudini Hospital during January-June 2005 were asked why they had come to the hospital. Reasons for non-compliance with referral were analyzed on a continual basis and contributed to fine-tuning of the system of facilitated referral. Randomly-selected parents of 162 newborns who were referred for care but did not comply with referral during the same period mentioned above were interviewed regarding their reasons for non-compliance. Structured brief questionnaires were used in both instances.

## RESULTS

### Functioning of the referral system

Results of the second adequacy survey conducted in September 2005 showed that 72.5% of the recently-delivered women had attended at least one antenatal care visit, and 87.6% had received at least one antenatal BNCP visit by a CHW.

Table 1 shows the trends between April 2004 and September 2005 in assessment, referral, and admission of sick newborns in the study intervention arm. Figure 1 presents data for the entire period. Although the project staff judged that the CHWs succeeded in identifying most pregnant women, the CHWs documented deliveries for only 3,354 (74.4%) of the 4,508 women estimated to have their due date during this period. The primary reason for this lower-than-expected figure was that significant numbers of women, particularly primigravidae, migrated at some point prior to delivery to live in another household located outside the intervention arm of the study. In most cases, they left the household of their husband's family (*shashurbari*) to reside in their natal home (*baperbari*), a phenomenon also observed in other sites in Bangladesh (20). Of 3,354 women who did not change residence, 3,228 (96.2%) received at least one home-visit by the CHW during the first 28 days of life of the baby.

**Table 1.** Trends over time in assessment, referral, and admission of sick newborns in Mirzapur sub-district, Tangail district, Bangladesh between April 2004 and September 2005 (*indicates that figures are also displayed in Fig. 1)

Variable and statistical assessment of trends	Apr-Jun 2004	Jul-Sep 2004	Oct-Dec 2004	Jan-Mar 2005	Apr-Jun 2005	Jul-Sep 2005	Total Apr 2004-Sept 2005*
Assessment and referral of sick newborns in the community by CHWs							
Home-deliveries	417	574	870	541	433	519	3,354*
CHW-visit within first 28 days	324	443	862	659	436	504	3,228*
Newborns assessed as sick	134	156	190	84	111	119	794*
Referred to Kumudini Hospital	115	148	161	71	87	102	684*
Percentage of sick newborns who were referred	85.8	94.9	84.7	84.5	78.4	85.7	86.1
Chi-square for linear trend=NS							
Arrival and admission at outpatient/emergency department of Kumudini Hospital							
Neonates reaching Kumudini Hospital OPD/emergency after self-referral or referral by CHWs	89	146	211	128	159	188	921*
Neonates reaching Kumudini OPD/emergency after self-referral	25	55	82	75	95	111	443*
Neonates reaching Kumudini Hospital OPD/emergency with CHW referral slip	64	91	129	53	64	77	478*
Chi-square for linear trend=43.5, df=1, p<0.00001	71.9	62.3	61.1	41.4	40.3	41.0	51.9
Percentage of newborns referred by CHWs who arrived at Kumudini Hospital OPD/emergency	55.7	61.5	80.1	74.6	73.6	75.5	69.9
Chi-square for linear trend=12.97, df=1, p=0.00032							

CHW=Community health worker;

df=Degree of freedom;

NS=Not significant;

OPD=Outpatient department

The CHWs identified 794 newborns as sick during this period, excluding newborns with feeding problems. Of these, 684 (86.1%) were referred by the CHWs to the Kumudini Hospital for care. The proportion of sick newborns who were referred stayed essentially constant between April 2004 and September 2005 (non-significant chi-square test for linear trend). Newborns arriving from households located in the intervention arm could either (a) have been referred by the CHW after she examined the child during a home-visit and issued a referral slip, or (b) the family could have taken the decision to seek care directly without first contacting the CHW, a situation we refer to as self-referral. Overall, of the 921 newborns arriving at the outpatient or emergency departments of the Kumudini Hospital during this period, 443 (48.1%) arrived after self-referral, and 478 (51.9%) arrived with a referral slip issued by the CHWs. The proportion of all cases who arrived with a referral slip declined continuously during this period (Row 8 of Table 1), from 71.9% at the beginning of implementation between April and June 2004 to 41.0% between July and September 2005, a trend which was highly significant (chi-square for linear trend=43.5, df=1, p<0.00001).

Compliance with referral to the Kumudini Hospital by the CHWs increased from 55.7% during the first three-month period of implementation to 80.1% during the third three-month period of implementation (Row 9 of Table 1) and was thereafter maintained at 75–80%. The overall trend was significant (p=0.00032).

### Interviews on factors affecting compliance

Eighty-four parents arriving in the Outpatient Department of the Kumudini Hospital were asked why they had come to the hospital. They mentioned (multiple responses allowed) that the CHWs advised them to visit the Kumudini Hospital because treatment was available (65.5%), treatment was of high quality (34.5%) and free of charge (21.4%), the hospital was the nearest one to their home (2.4%), and other reasons (4.8%).

Parents of 162 newborns who were referred for care but did not comply with referral were interviewed. The reasons cited for non-compliance (multiple responses allowed) included: nobody was available to accompany the child (and the mother) to the health facility (24.7%); the child was given a traditional treatment instead (19.1%); bad weather or general strikes (17.9%); the family disliked hospital treatment (12.3%); symptoms resolved on their own (7.4%); unwillingness of the family or the TBA to refer the baby for other reasons (6.2%); and other issues (12.3%), such as illness of the mother; the child was too young to be taken for outside care; and lack of transport. Although distance was a commonly-cited reason for failure to seek care from the health facilities, the proportion of cases referred by the CHWs in the farthest unions in the intervention arm, Warsi (2 hours away), Ajgana (1.5 hours away), and Bahuria (1.5 hours away) were at approximately the same level (79.3%, 80.6%, and 82.7% respectively) as the closest unions that were 0.5 hours away from the Kumudini Hospital, Banail (87.9%), and Bhatgram (81.6%).

### Evaluation of trends in care-seeking

Table 2 demonstrates the changes in care-seeking/self-referral to the Kumudini Hospital and other providers between the baseline survey (January 2003) and the first (January 2005) and the second (September 2005) adequacy survey. Caution is necessary in assigning significance to the observed trends as the two adequacy surveys are based on much smaller samples than the baseline survey. At the time of the baseline household survey in January 2003, there was no significant difference between the intervention arm and the comparison arm of the study in the proportion of sick newborns who were taken outside the home for care from any qualified provider (including Kumudini Hospital), to the Kumudini Hospital specifically, or to an unqualified provider, most commonly an unlicensed ‘village doctor’.

**Table 2.** Trends in care-seeking for sick newborns from Kumudini Hospital and other providers based on population-based household surveys

Measures of care-seeking	Baseline survey: January 2003		First adequacy ssurvey: January 2005		Second adequacy survey: September 2005	
	Intervention arm	Comparison arm	Intervention arm	Comparison arm	Intervention arm	Comparison arm
Reported sickness among newborns in the sample						
Total number of newborns in the sample	2,053	2,290	523	550	520	548
Number of newborns reported to have been sick (%)	818 (37.5)	780 (37.6)	255 (48.8)	279 (50.7)	207 (39.8)	257 (46.9)
Reported care-seeking outside home						
Proportion of sick newborns reported to have been given any care in or outside home	92.9	93.7	93.3	93.2	93.2	95.3
Proportion of sick newborns for whom care was sought from outside home	66.1	66.4	82.8	81.4	82.6	77.4
Reported care-seeking from qualified providers (Fig. 2)						
Proportion of sick newborns for whom care was sought from qualified providers	31.2	29.6	55.7	38.4	60.4	33.9
Odds ratio for care from qualified providers, intervention vs comparison (95% CI)	1.08 (0.86–1.34) (p=0.499)		2.02 (1.41–2.90) (p<0.0001)		2.98 (2.00–4.44) (p<0.0001)	
Chi-square trend test for care from qualified providers, intervention group	Chi-square for linear trend=80.13, df=1, p<0.00001					
Chi-square trend test for care from qualified providers, comparison group	Chi-square for linear trend=3.42, df=1, p=0.065					
Reported care-seeking from Kumudini Hospital (Fig. 3)						
Proportion of sick newborns for whom care was sought from Kumudini Hospital	17.9	17.6	42.0	21.9	46.4	23.0
Odds ratio for care from Kumudini Hospital, intervention vs comparison (95% CI)	1.02 (0.78–1.33) (p<0.882)		2.58 (1.74–3.84) (p<0.0001)		2.90 (1.91–4.41) (p<0.0001)	
Chi-square trend test for care from Kumudini Hospital, intervention Group	Chi-square for linear trend=93.40, df=1, p<0.00001					
Chi-square trend test for care from Kumudini Hospital, comparison Group	Chi-square for linear trend=4.47, df=1, p=0.035					
Reported care-seeking from unqualified providers (Fig. 4)						
Proportion of sick newborns for whom care was sought from unqualified providers	66.7	67.9	49.8	66.0	36.7	65.0
Odds ratio for care from unqualified providers, intervention vs comparison (95% CI)	0.95 (0.76–1.17) (p<0.609)		0.51 (0.36–0.74) (p=0.0002)		0.31 (0.21–0.47) (p<0.0001)	
Chi-square trend test for care from unqualified providers, intervention group	Chi-square for linear trend=71.01, df=1, p<0.00001					
Chi-square trend test for care from unqualified providers, comparison group	Chi-square for linear trend=0.90, df=1, p=0.34					

CI=Confidence interval;

df=Degree of freedom

**Fig. 2.**
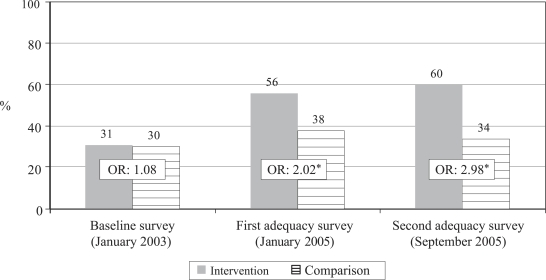
Trend in proportion of sick newborns for whom care was sought from qualified providers

**Fig. 3.**
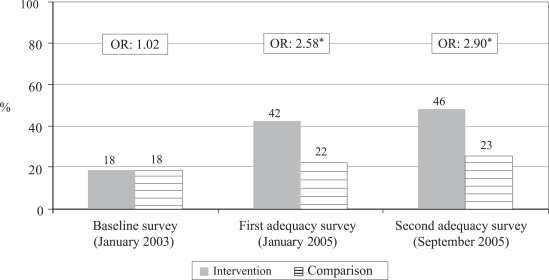
Trend in proportion of sick newborns for whom care was sought from Kumudini Hospital

**Fig. 4.**
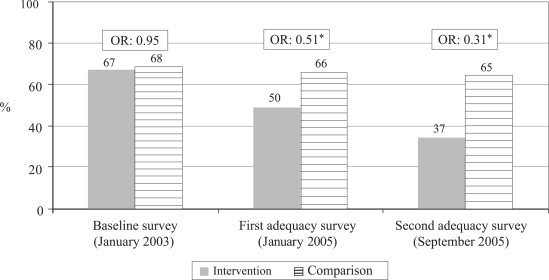
Trend in proportion of sick newborns for whom care was sought from unqualified providers

Between the time of the baseline and the first adequacy survey, there was a significant increase in care-seeking in both intervention and comparison arms of the study (Row 4 of Table 2). This difference could be due to both increased awareness of the newborn's health problems and/or changes in how the survey was administered. Figure 2 demonstrates that there was a highly significant (p<0.00001) increase in the proportion of families that sought care from qualified providers for sick newborns in the intervention arm (Fig. 2 and Rows 5 and 7 of Table 2) and a non-significant increase in the comparison arm (Rows 5 and 8). Figure 3 shows that care-seeking for sick newborns specifically from the Kumudini Hospital increased significantly in both intervention and comparison arms, but the increase was of a much greater magnitude in the intervention arm (Fig. 3 and Row 9–12 of Table 2). Finally, Figure 4 demonstrates that the proportion of families who sought care from unqualified providers, such as unlicensed village doctors, remained unchanged in the comparison arm, but declined significantly in the intervention arm (Fig. 4 and Row 13–16 of Table 2).

*p<0.0001; Chi-square for linear trend (intervention) 80.13 (p<0.00001)
OR=Odds ration

*p<0.0001; Chi-square for linear trend (intervention) 93.4 (p<0.00001)
OR=Odds ratio

*p<0.0001; Chi-square for linear trend (intervention) 71.01 (p<0.00001)
OR=Odds ratio

## DISCUSSION

Significant reductions in neonatal mortality must be made to reach the Millennium Development Goal for under-five mortality. One key to reducing neonatal mortality is to ensure that sick newborns are assessed and treated quickly either in the home (22, 23) or in a health facility. Practices of postpartum confinement of mothers and newborns found in many cultures, difficulties in transport, and patterns of household decision-making are among the factors that can delay or prevent care-seeking outside the home. Data presented in this paper demonstrate that it is possible to achieve high rates of care-seeking from hospitals or other qualified providers and to significantly decrease care-seeking from unqualified providers, even in a low-income rural area where all these factors are present. The trends observed in the intervention arm included increased care-seeking, increased proportion of sick newborns arriving at the Kumudini Hospital after self-referral rather than referral by a CHW, increased compliance after referral by the CHWs, increased care-seeking from the qualified providers (Fig. 2) and from the Kumudini Hospital (Fig. 3), and decreases in care-seeking from the unqualified providers (Fig. 4). In the comparison arm, an increase in care-seeking from the Kumudini Hospital was also observed, but the proportions seeking care from the qualified and unqualified providers did not change significantly.

Reasons for the increasing trend in the proportion of cases in the intervention arm arriving at the Kumudini Hospital after self-referral have not yet been fully elucidated, but include increasing awareness of the signs of illness in newborns and experience with the services offered by the Kumudini Hospital. There also appeared to be some cases where the CHWs judged that the newborns did not require referral, but the family decided to seek care from the Kumudini Hospital anyway. In a number of these cases, the child had physiologic jaundice, a condition which the family felt warranted further investigation, despite reassurances provided by the CHWs. The project investigators are currently investigating this situation further.

Substantial increases in referral compliance for newborn illness were likely related to (a) education of families on danger signs by the CHWs; (b) active surveillance for illness by the CHWs during routine postnatal home-visits; (c) facilitated referral by the CHWs, including counselling, use of referral slips along with improved linkages between community and hospital; (d) incentives for labour/birth notification; (e) enhanced capacity at the referral-care centre to manage sick newborns; and (f) availability of subsidized treatment. Sustained community-level education enhanced the empowerment of families towards decision-making for self-referral.

In low-income countries with high rates of neonatal mortality, sick newborns can either be treated presumptively in the home (22, 23) or referred to health facilities. This paper demonstrates that it is possible to achieve high rates of compliance with referral, but to do this requires an extensive infrastructure of CHWs or other community contact persons (8) to assess newborns and facilitate referrals. Alternatively, an increased emphasis could be placed on community mobilization, education on danger signs, and facilitation of self-referral, perhaps with similar results. This paper is one of the only studies to provide data on levels of referral compliance achieved through facilitated referral (12, 14). Whether the emphasis is on treatment in facilities or treatment in the community, substantial investments will need to be made in the creation of demand with the community, community and family education, and facilitation of referral.

## ACKNOWLEDGEMENTS

This study was supported primarily through the generous support of the Infectious Disease Initiative of the Wellcome Trust–Burroughs Wellcome Fund. Additional support was provided by the Department for International Development (DFID), UK; the United States Agency for International Development, Office of Health, Infectious Diseases, and Nutrition, Global Bureau through the Global Research Activity Cooperative Agreement (No. GHS-A-00-03-00019-00); the Government of Bangladesh (Improved Health for the Poor); and Save the Children-USA through a grant from the Bill and Melinda Gates Foundation.

The authors thank the study participants in Mirzapur upazila, Bangladesh, who were generous with their time and patience with interviewers through the several rounds of interviews.

## References

[1] Amarasiri de Silva MW, Wijekoon A, Hornik R, Martines J (2001). Care seeking in Sri Lanka: one possible explanation for low childhood mortality. Soc Sci Med.

[2] Black RE, Morris SS, Bryce J (2003). Where and why are 10 million children dying every year?. Lancet.

[3] Barnes-Josiah D, Myntti C, Augustin A (1998). The “three delays” as a framework for examining maternal mortality in Haiti. Soc Sci Med.

[4] Koblinsky MA, Campbell O, Heichelheim J (1999). Organizing delivery care: what works for safe motherhood?. Bull World Health Organ.

[5] Ahluwalia IB, Schmid T, Kouletio M, Kanenda O (2003). An evaluation of a community-based approach to safe motherhood in northwestern Tanzania. Int J Gynaecol Obstet.

[6] Ganatra BR, Coyaji KJ, Rao VN (1998). Too far, too little, too late: a community-based case-control study of maternal mortality in rural west Maharashtra, India. Bull World Health Organ.

[7] Martey JO, Djan JO, Twum S, Browne EN, Opoku SA (1998). Referrals for obstetrical complications from Ejisu district, Ghana. West Afr J Med.

[8] Nwakoby B, Akpala C, Nwagbo D, Onah B, Okeke V, Chukudebelu W (1997). Community contact persons promote utilization of obstetric services, Anambra State, Nigeria. The Enugu PMM Team. Int J Gynaecol Obstet.

[9] Murray SF, Pearson SC (2005). Maternity referral systems in developing countries: current knowledge and future research needs. Soc Sci Med.

[10] Sibley L, Sipe TA, Koblinsky M (2004). Does traditional birth attendant training improve referral of women with obstetric complications: a review of the evidence. Soc Sci Med.

[11] Kalter HD, Schillinger JA, Hossain M, Burnham G, Saha S, de Wit V (1997). Identifying sick children requiring referral to hospital in Bangladesh. Bull World Health Organ.

[12] Kalter HD, Salgado R, Moulton LH, Nieto P, Contreras A, Egas ML (2003). Factors constraining adherence to referral advice for severely ill children managed by the Integrated Management of Childhood Illness approach in Imbabura Province, Ecuador. Acta Paediatr.

[13] Font F, Quinto L, Masanja H, Nathan R, Ascaso C, Menendez C (2002). Paediatric referrals in rural Tanzania: the Kilombero district study—a case series. BMC Int Health Hum Rights.

[14] Winch PJ, Gilroy KE, Wolfheim C, Starbuck ES, Young MW, Walker LD (2005). Intervention models for the management of children with signs of pneumonia or malaria by community health workers. Health Policy Plan.

[15] Macintyre K, Hotchkiss DR (1999). Referral revisited: community financing schemes and emergency transport in rural Africa. Soc Sci Med.

[16] Sibley L, Caleb-Varkey L, Upadhyay J, Prasad R, Saroha E, Bhatia N (2005). Recognition of and response to postpartum hemorrhage in rural northern India. J Midwif Womens Health.

[17] Terra de Souza AC, Peterson KE, Andrade FM, Gardner J, Ascherio A (2000). Circumstances of post-neo-natal deaths in Ceara, Northeast Brazil: mothers' health care-seeking behaviors during their infants' fatal illness. Soc Sci Med.

[18] Sutrisna B, Reingold A, Kresno S, Harrison G, Utomo B (1993). Care-seeking for fatal illnesses in young children in Indramayu, West Java, Indonesia. Lancet.

[19] de Zoysa I, Bhandari N, Akhtari N, Bhan MK (1998). Careseeking for illness in young infants in an urban slum in India. Soc Sci Med.

[20] Winch PJ, Alam MA, Akther A, Afroz D, Ali NA, Ellis AA (2005). Local understandings of vulnerability and protection during the neonatal period in Sylhet district, Bangladesh: a qualitative study. Lancet.

[21] Reddy MH, Bang AT (2005). How to identify neonates at risk of death in rural India: clinical criteria for the risk approach. J Perinatol.

[22] Bang AT, Bang RA, Stoll BJ, Baitule SB, Reddy HM, Deshmukh MD (2005). Is home-based diagnosis and treatment of neonatal sepsis feasible and effective? Seven years of intervention in the Gadchiroli field trial (1996 to 2003). J Perinatol.

[23] Bang AT, Bang RA, Baitule SB, Reddy MH, Deshmukh MD (1999). Effect of home-based neonatal care and management of sepsis on neonatal mortality: field trial in rural India. Lancet.

